# The Dark Side of Authenticity: Feeling “Real” While Gambling Interacts with Enhancement Motives to Predict Problematic Gambling Behavior

**DOI:** 10.1007/s10899-014-9460-7

**Published:** 2014-05-10

**Authors:** Jamey J. Lister, Michael J. A. Wohl, Christopher G. Davis

**Affiliations:** 1 Center for Gambling Studies, School of Social Work, Rutgers University, 536 George Street, New Brunswick, NJ 08901 USA; 2 Department of Psychology, Carleton University, 1125 Colonel By Drive, B550 Loeb Building, Ottawa, ON K1S 5B6 Canada

**Keywords:** Authenticity, Enhancement, Pathology, Gambling, Well-being, Sports betting

## Abstract

Engaging in activities that make people feel authentic or real is typically associated with a host of positive psychological and physiological outcomes (i.e., being authentic serves to increase well-being). In the current study, we tested the idea that authenticity might have a dark side among people engaged in an addictive or risky behavior (gambling). To test this possibility, we assessed gamblers (*N* = 61) who were betting on the National Hockey League playoff games at a sports bar. As predicted, people who felt authentic when gambling reported behavior associated with problem gambling (high frequency of betting) as well as problematic play (a big monetary loss and a big monetary win). Moreover, such behavior and gambling outcomes were particularly high among people who were motivated to gamble for the purpose of enhancement. The interaction of feeling authentic when betting and gambling for purposes of enhancing positive emotions proved especially troublesome for problematic forms of play. Implications of authenticity as a potential vulnerability factor for sports betting and other types of gambling are discussed.

## Introduction

Well-being is typically enhanced when people feel they are genuine in their day-to-day functioning—when behavior is consistent with values and goals (Goldman and Kernis 2002; Wood et al. 2008). In psychological terms, people who function in this way are said to be high in authenticity—a manner of being that is one of the strongest predictors of well-being (Wood et al. 2008). For example, Sheldon et al. (1997) found that feelings of authenticity promoted self-esteem and protected against depression and anxiety. In this light, authenticity is a way of being that people should strive to achieve and maintain.

We suggest, however, that there might be contexts in which feeling authentic will have negative consequences. Specifically, if a person feels authentic when engaging in an addictive behavior, a continuation of that addictive behavior is likely. For example, for some people the intensity of gambling helps to organize and focus their mental state serving to make them “feel real” (Sanger 2003)—a central feature of authenticity. The authentic feeling gambling provides should result in increasing attractiveness to and engagement in wagering behavior. Unfortunately, symptoms of gambling pathology tend to increase alongside the frequency of play (Stewart and Zack 2008). In the current study, we directly test the idea that feelings of authenticity might have a dark side. In particular, we examine the possibility that gamblers who feel authentic when they gamble may be a high-risk population—one that plays excessively and thus is especially likely to have experienced both the big highs and lows of gambling. These highs and lows should manifest in the experience of a very large win as well as a very large loss—as are bound to occur with frequent play (LaBrie et al. 2007).

We also examine whether feeling authentic is particularly problematic for gamblers who are enhancement-motivated, i.e., people who are motivated by the positive reinforcing aspects of gambling (e.g., feeling good). The combination of feeling authentic while gambling and gambling in order to enhance positive emotion is likely a dangerous cocktail that drives the gambler to win big, but in the process experience big losses.

### Authenticity

People seek authenticity in their life and those who experience feelings of authenticity tend to report higher levels of psychological and physical well-being (see Goldman and Kernis 2002; Sheldon et al. 1997; Wood et al. 2008). In this manner, feeling that one’s behavior is authentic may provide a sense that one’s life is fulfilling and meaningful (Gecas 1994). Behavior associated with such feelings might be particularly resistant to change; those who feel authentic whilst gambling may not recognize their behavior as problematic, or if they do, may be reluctant to give up something that is for them life-affirming. Akin to other thrill-seekers (see Self et al. 2007), we suspect that people who feel authentic whilst gambling may continually put themselves at risk (via continued engagement) despite experiencing negative consequences that stem from that behavior.

In the current paper, we test the idea that people who feel authentic whilst sports betting are likely to be frequent bettors with associated negative outcomes (i.e., significant monetary losses). In this way, gambling may offer a forum to express what the gambler perceives to be his or her “true personality” (J. Beck, personal communication, August 10, 2010). This contention is in line with Freud’s distinction between ego-syntonic and ego-dystonic behaviors. Specifically, Freud argued that ego-syntonic behavior was felt to be acceptable and/or consistent with one’s self-conception, while ego-dystonic behavior was in conflict or inconsistent with the self (Freud 1961). To date, however, the possible association between authenticity and problematic gambling behavior remains theoretical and is thus deserving of empirical attention.

### Gambling Motives as Moderator of the Authenticity Effect on Gambling

Problematic gambling behavior has also been conceptualized as ego-syntonic because it satisfies the gambler’s pleasure seeking motivations (el-Guebaly et al. 2012). Indeed, gambling fulfills psychological needs for fun and excitement (Lee et al. 2007). According to Stewart and Zack (2008), the gambler is enhancement-motivated to the extent that (s)he plays to feel good. Consistent with el-Guebaly et al.’s (2012) ego-syntonic understanding of problematic play, Stewart and Zack (2008) found that high levels of enhancement-motivation were positively associated with gambling frequency and symptoms of problem gambling. Thus, in addition to feeling authentic whilst playing, enhancement motives are likely a key predictor of problematic gambling behaviors and associated outcomes.

Herein we suggest that although enhancement motives and feelings of authenticity may both independently predict gambling behavior, there is reason to believe that in combination they may be particularly toxic. A person who gambles because (s)he feels authentic whilst doing so, but is motivated to play for the social connections it provides (i.e., social motivated gamblers; see Stewart and Zack 2008) is only going to play when the opportunity for interactions with others is possible. Conversely, the enhancement-motivated gambler can feel good when gambling alone as well as with others. Additionally, the enhancement-motivated gambler who does feel authentic whilst gambling is likely to search for (and engage in) activities that facilitate feelings of authenticity. When a gambler feels authentic whilst playing and is motivated to enhance positive emotions s(he) will likely play frequently in order to feel authentic and engagement in any type of gambling provides the opportunity to enhance. Moreover, frequent play should manifest in reports of a large biggest win and, ominously, a large biggest loss—with greater frequency of play there is more opportunity for big wins and big losses (see LaBrie et al. 2007; Turner et al. 2006). To this end, we examine enhancement-motivations as a moderator of the hypothesized direct effect of authenticity on problematic gambling behaviors and outcomes.

## Method

### Participants

The inclusion criteria were kept broad as a means of keeping the sample as representative as possible; participants only needed to have bet on sports in the past year to be eligible for enrollment. Sixty-nine participants were recruited during their patronage at a local sports bar, eight of whom were excluded from analyses because they failed inclusion criteria or failed to follow instructions. After exclusions, sixty-one (55 Male, 6 Female) patrons of a popular sports bar in a major metropolitan area of Ontario participated in the study. Participants ranged in age from 20 to 76 years, with a mean age of 35.18 years (*SD* = 11.96). All participants received a $5 gift card towards the establishment for taking part in the study.

### Procedures and Measures

Participants were recruited to complete our pencil-and-paper questionnaire just prior to and during a National Hockey League playoff game in a sports bar in a Canadian city. All participants read an informed consent and were assured their responses would be anonymous. Participants were then allowed to complete the questionnaire at either the study table or their own table, provided they could do so without their responses being influenced by others. Following the completion of the questionnaire, all participants were fully debriefed.

#### Authenticity

Participants completed a four-item measure that assessed feelings of authenticity during sports betting (adapted from Fleeson and Wilt 2010; α = .75). These items were: “When I am betting on sports, I feel like my true self,” “I feel authentic when I am betting on sports,” “I feel very comfortable with myself when I bet on sports,” and “I feel like I am putting on an act when I bet on sports (reverse-scored).” The authenticity scale was anchored at 1 (*strongly disagree*) and 7 (*strongly agree*).

#### Motivation for Gambling

The Gambling Motives Questionnaire (GMQ; Stewart and Zack 2008) was modified slightly to assess the gambler’s motivation for sports betting. Of particular interest to this study was a three-item subscale assessing enhancement motives for betting (α = .70). These items were: “I bet on sports because it’s exciting,” “I bet on sports because it’s fun,” and “I bet on sports because it makes me feel good.” The enhancement subscale items were anchored at 1 (*strongly disagree*) and 7 (*strongly agree*).

#### Problematic Gambling Behavior and Outcomes

All participants completed a demographic form regarding their gambling behavior, three of the measures functioned as the dependent variables of interest to assess the level of problematic gambling behavior and outcomes. Betting frequency was assessed with an item that asked about the frequency with which they bet on sports. Response options ranged from 1 (*rarely*) to 4 (*all the time*). We also asked participants to indicate their biggest win and biggest loss from sports betting. Biggest win ranged from $0 to $24,000 (median = $100), and biggest loss ranged from $3 to $800 (median = $25). Because the distribution of responses to both of these questions was positively skewed, we normalized the distributions by log transforming each win and loss. All reported results are with the log-transformed variables.

## Results

Not surprisingly, the more frequently people reported betting on sporting events, the greater was their biggest win (*r* = .68, *p* < .001) and their biggest loss (*r* = .47, *p* < .001). Biggest win and biggest loss were also positively correlated, *r* = .61, *p* < .001. Feelings of authenticity were correlated significantly with gambling for enhancement, *r* = .46, *p* < .001, and both authenticity and gambling for enhancement scores were correlated significantly and positively with frequency of betting and biggest loss. Authenticity was correlated significantly and positively with biggest win (*r* = .46, *p* < .001); gambling for enhancement was not (*r* = .19, *p* = .15). Correlations among study variables are provided in Table 1.

**Table 1:** Correlations among Measured Variables with Mean and Standard Deviation on the Diagonal

	1	2	3	4	5
1 Authenticity	4.60 (1.38)				
2 Betting frequency	.51***	2.00 (.95)			
3 Biggest win (log)	.46***	.68***	4.69 (2.04)		
4 Biggest loss (log)	.31*	.47***	.61***	3.59 (1.27)	
5 Enhancement motives	.46***	.39**	.19	.28*	4.70 (1.22)

* *p* < .05, ** *p* < .01, *** *p* < .001

**Fig. 1:**
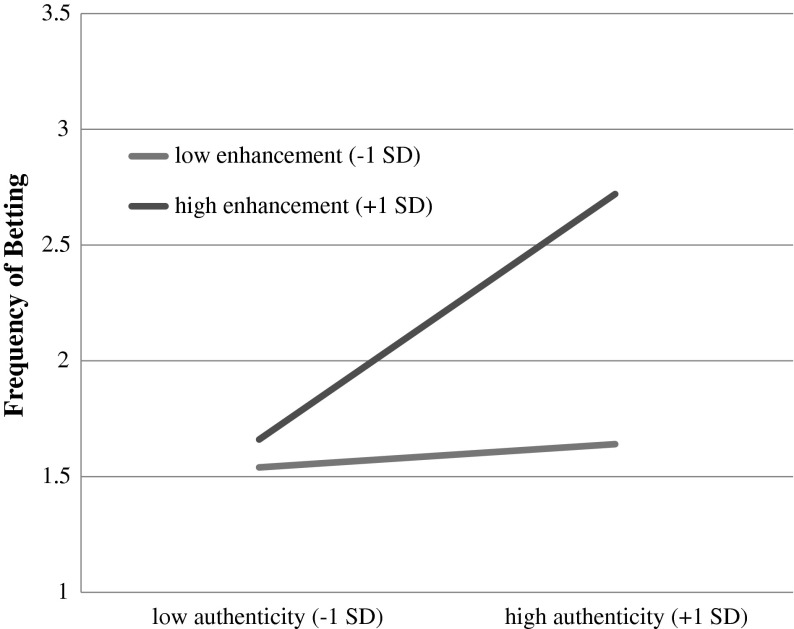
Moderation model with authenticity as the independent variable, enhancement motives as the moderator, and frequency of betting as the dependent variable

**Fig. 2:**
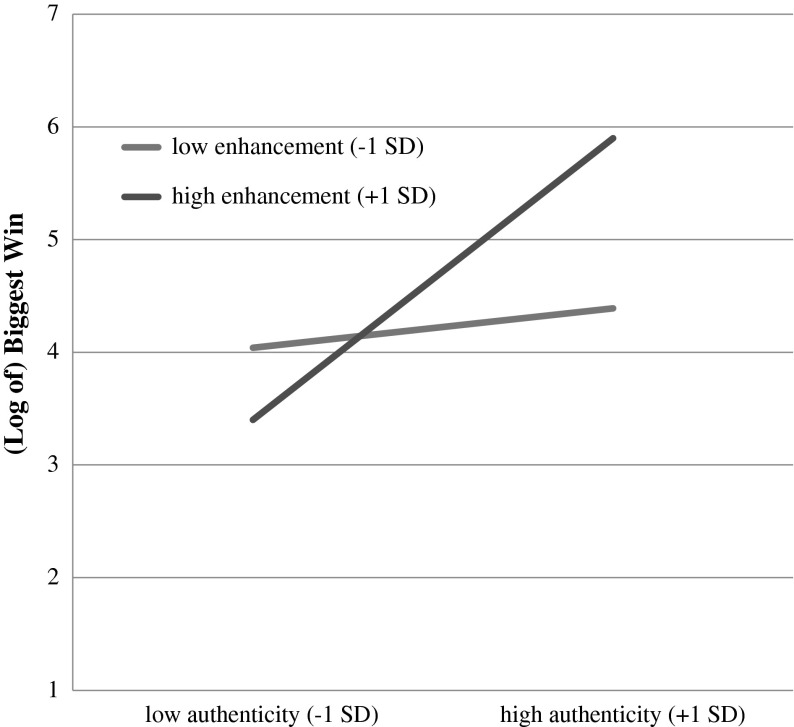
Moderation model with authenticity as the independent variable, enhancement motives as the moderator, and biggest win as the dependent variable

**Fig. 3:**
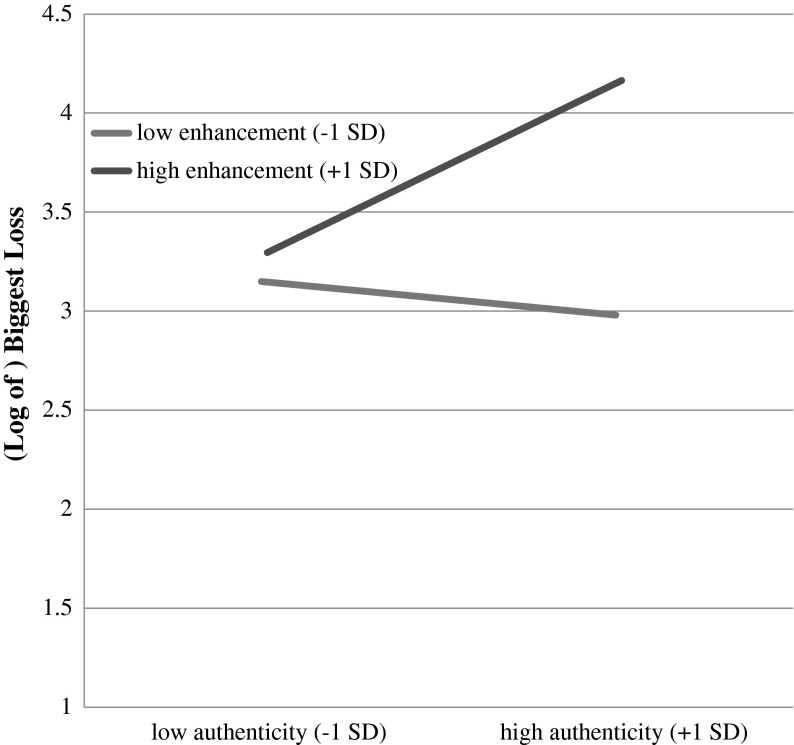
Moderation model with authenticity as the independent variable, enhancement motives as the moderator, and biggest loss as the dependent variable

Three multiple regressions were conducted to assess the unique and interaction effects of authenticity (mean-centered) and gambling for enhancement (mean-centered) on frequency of betting, biggest loss, and biggest win (Figs. 1, 2, 3). The first of these analyses, predicting frequency of betting, indicated a significant effect of authenticity (*b* = .21, *se* = .09, *t* = 2.45, *p* = .02), a main effect of gambling for enhancement (*b* = .25, *se* = .10, *t* = 2.54, *p* = .02), and a significant authenticity X gambling for enhancement interaction (*b* = .14, *se* = .05, *t* = 2.82, *p* = .001; *R* ^*2*^ = .38, *F* (3, 57) = 11.53, *p* < .001). Simple slopes analysis of the interaction term indicated that when enhancement motives were low (−1 *SD*), the effect of authenticity was negligible (*b*_authenticity_ = .03, *se* = .12, *p* = .78). However, when enhancement motives were at the mean or high (+1 *SD*), the effect of authenticity on betting frequency was significant and positive (when enhancement was at the mean, *b*_authenticity_ = .20, *se* = .09, *t* = 2.46, *p* = .02; when enhancement was +1 *SD*, *b*_authenticity_ = .39, *se* = .09, *t* = 4.40, *p* < .001).

The second regression, on biggest win, yielded a significant main effect of authenticity (centered; *b* = .51, *se* = .20, *t* = 2.59, *p* = .01), a non-significant effect for gambling for enhancement (centered; *b* = .17, *se* = .23, *t* = .74, *p* = .46), and a significant authenticity X gambling for enhancement interaction (*b* = .31, *se* = .12, *t* = 2.70, *p* = .009; *R* ^*2*^ = .30, *F* (3, 56) = 7.98, *p* < .001). Simple slopes analysis on the interaction term indicated a similar pattern of results to those for betting frequency. When enhancement motives were low (−1 *SD*), the effect of authenticity was trivial (*b*_authenticity_ = .12, *se* = .28, *t* = .43, *p* = .67). But when enhancement motives were at the mean or above (+1 *SD*), the effect of authenticity was positive and significant (when enhancement was at the mean, *b*_authenticity_ = .51, *se* = .20, *t* = 2.58, *p* = .01; when enhancement was +1 *SD*, *b*_authenticity_ = .89, *se* = .20, *t* = 4.47, *p* < .001).

The third regression, on biggest loss, yielded neither a main significant effect of authenticity (centered; *b* = .13, *se* = .14, *t* = .98, *p* = .33) nor gambling for enhancement (centered; *b* = .29, *se* = .16, *t* = 1.83, *p* = .07). There was, however, a significant interaction of authenticity by gambling for enhancement (*b* = .16, *se* = .08, *t* = 1.98, *p* = .05; *R* ^*2*^ = .18, *F* (3, 55) = 3.93, *p* = .01). Simple slopes analysis on the interaction term was similar to that observed for the other measured variables. The effect of authenticity was trivial at −1 *SD* of enhancement motives (*b*_authenticity_ = −.06, *se* = .18, *t* = −.30, p = .76). The effect of authenticity was also trivial at the mean of enhancement motives (*b*_authenticity_ = .13, *se* = .13, *t* = .98, *p* = .33). At + 1 SD of enhancement motives, however, the effect of authenticity was positive and significant (*b*_authenticity_ = .31, *se* = .14, *t* = 2.30, *p* = .04).

## Discussion

In the current research, we examined the heretofore unexamined negative consequences of feeling authentic when engaged in an addictive behavior. We predicted and found that feeling authentic whilst sports betting predicted frequency of betting as well as outcomes associated with gambling pathology—large monetary wins and losses. This exploration of authenticity as a vulnerability factor for risky behavior provides preliminary evidence for the counterintuitive role that a conventionally understood protective factor can play. In sum, authenticity may serve to undermine well-being in specific contexts.

We also showed that enhancement motives predict frequency of betting as well as outcomes of problematic behavior (i.e., significant monetary loss and gain) alongside authenticity. Not only do these feelings of authenticity and enhancement motives uniquely predict problematic gambling behavior, they appear to interact. Specifically, we showed that the combination of feeling authentic and being motivated by the positively reinforcing aspects of betting (i.e., being enhancement-motivated) is a dangerous cocktail for the gambler. In this light, we found evidence that an interaction of ego-syntonic personality (being authentic) and behavior (positive emotion enhancement) may be particularly troublesome.

Our findings build on prior work that has found counterintuitive associations between conventionally understood protective factors and vulnerability in the gambling domain. Prior research, for instance, has found heightened risk of problematic forms of play among optimists as well as those who set high achievement gambling-oriented goals (Gibson and Sanbonmatsu 2004; Lister et al. 2014). The exploration of previously understood protective factors having risk-associated qualities is still in its infancy, but the current results suggest additional research is warranted. We suspect authenticity might play an important role in the development and maintenance of maladaptive behaviors other than gambling. Indeed, anyone who engages in risky behavior because they feel authentic when doing so is likely to experience some negative outcomes. The point here is that harnessing authenticity should not be viewed as a panacea for the improvement of psychological well-being.

Some caveats of the current research should be noted. First, participants in our research may not be representative of all sports bettors, gamblers who play other types of games (e.g., slot machines), or gamblers who play for different motivations (e.g., coping or social motives). In the current study, the sample of participants was predominately male. Given that males and females are differentially motivated to play—men typically gamble to enhance positive emotion, whereas females are typically motivated to gamble to cope or for the social connections gambling provides (See Stewart and Zack 2008)—and that women tend not to sports bet (instead gravitating to non-strategic games like slot machines; see Ledgerwood and Petry 2006; Potenza et al. 2001) the observed effects might be less pronounced among females.

Second, people (male or female) who are motivated to gamble for social reasons tend to have low rates of disordered gambling (Stewart and Zack 2008). If socially motivated gamblers had been examined in the current study, they would likely have reported a low betting frequency, regardless of feelings of authenticity whilst sports betting.

Third, people (male or female) who prefer to play non-strategic games tend not to be motivated to enhance positive emotion (Bonnaire et al. 2009). Thus, they would likely be absent from the current sample of sports bettors. Nonetheless, future research should investigate the associations among feelings of authenticity, motivations to gamble other than enhancement, and gambling behavior as well as outcomes, especially as they relate to female gamblers and motivations for gamblers playing non-strategic gambling games.

Lastly, although the method employed has substantial external validity due to our method of recruitment (assessing gamblers whilst betting), it is possible some participants were distracted by the hockey game they were watching and thus full attention was not given to the questionnaire they were completing. To the extent that it introduces random noise, distracted responding is apt to underestimate effects.

## Conclusion

This project sought to explore the counterintuitive role that feelings of authenticity may have in a sample of sports bettors. Previous research has typically explored the protective relationship between authenticity and positive outcomes such as well-being. We examined whether in certain circumstances authenticity could underpin potentially harmful behavior. We found that feelings of authenticity and gambling for enhancement may place individuals at heightened risk for problematic gambling behavior and outcomes. These findings suggest a more nuanced understanding of authenticity, one that recognizes its potential dark side.
